# It’s all about the money? A qualitative study of healthcare worker motivation in urban China

**DOI:** 10.1186/s12939-017-0616-9

**Published:** 2017-07-07

**Authors:** Ross Millar, Yaru Chen, Meng Wang, Liang Fang, Jun Liu, Zhidong Xuan, Guohong Li

**Affiliations:** 1 Health Services Management Centre, University of Birmingham, Birmingham, UK; 2 Warwick Business School, University of Warwick, Coventry, UK; 3 School of Public Health, Shanghai Jiao Tong University School of Medicine, Shanghai, China; 4 Shihezi City People’s Hospital, Urumqi, China; 5 Institute of Social Medicine and Health Management, Henan University, Kaifeng, China; 6 Center for HTA, China Hospital Development Institute, Shanghai Jiao Tong University, Shanghai, China

**Keywords:** Motivation, Health workers, Renumeration, Trust relationships, Governance, Human resource management

## Abstract

**Background:**

China’s healthcare reform programme continues to receive much attention. Central to these discussions has been how the various financial incentives underpinning reform efforts are negatively impacting on the healthcare workforce. Research continues to document these trends, however, qualitative analysis of how these incentives impact on the motivation of healthcare workers remains underdeveloped. Furthermore, the application of motivational theories to make sense of healthcare worker experiences has yet to be undertaken.

**Methods:**

The purpose of our paper is to present a comparative case study account of healthcare worker motivation across urban China. It draws on semi structured interviews (*n* = 89) with a range of staff and organisations across three provinces. In doing so, the paper analyses how healthcare worker motivation is influenced by a variety of financial incentives; how motivation is influenced by the opportunities for career development; and how motivation is influenced by the day to day pressures of meeting patient expectations.

**Results:**

The experience of healthcare workers in China highlights how a reliance on financial incentives has challenged their ability to maintain the values and ethos of public service. Our findings suggest greater attention needs to be paid to the motivating factors of improved income and career development. Further work is also needed to nurture and develop the motivation of healthcare workers through the building of trust between fellow workers, patients, and the public.

**Conclusions:**

Through the analysis of healthcare worker motivation, our paper presents a number of ways China can improve its current healthcare reform efforts. It draws on the experience of other countries in calling for policy makers to support alternative approaches to healthcare reform that build on multiple channels of motivation to support healthcare workers.

## Background

China’s landmark healthcare reforms of 2009 continue to receive much attention [1, 2]. Defined as ‘one of the biggest health policy interventions in modern history in terms of both scale and scope’ [3], the reform programme has contained a range of measures that aim to achieve universal health coverage through affordable and accessible service provision [4–7].

Reforms such as the expansion of social insurance are reported to have led to benefits for the population by increasing access to services and lowering the cost of certain healthcare procedures. Yet many document the adverse consequences of these reform efforts in their association with high out of pocket payments, profit driven providers, and healthcare workers ‘trapped between social obligations and new economic incentives’ [3, 8]. Deteriorating doctor-patient relationships [9], defensive medicine practices [10], and physicians labelled as ‘private entrepreneurs’ [1] remain key policy challenges for the Chinese healthcare system.

At issue appears to be the performance related pay systems that healthcare workers are required to fulfil. Weng et al. [11], along with others [12], summarise how healthcare workers form part of a public sector performance-related pay (PRP) system where income is based on position in the organisational hierarchy, the achievement of performance benchmarking (e.g. passing annual appraisals), the performance of organisational units in which workers reside, and additional subsidies depending on particular environmental circumstances.

The aim of these financial incentives has been to motivate workers as earnings are related to performance. However, problems regarding misaligned incentives and unintended consequences of such arrangements have been documented [12], particularly in relation to healthcare worker motivation and morale. In China, low job satisfaction has been attributed to heavy workload, poor working conditions, and low income [13, 14]. Perceptions of inequity within the healthcare system have also been noted where financial rewards are perceived to be significantly lower in primary care compared with secondary and tertiary hospitals [15].

Reflecting on this worker dissatisfaction, Hung et al. [16] note the considerable room for improvement needed in China with regards to strengthening human resources, improving nonfinancial incentives, and delivering high quality care. Zhang et al. [4] call on policy-makers to pay greater attention to improving the financial remuneration for workers in a manner that will be perceived as equitable and motivating. A change to the current fee for service arrangements is also called for with alternative methods such as capitation payments and work-volume-based methods being recommended for allocating government subsidies to prevent profit seeking behaviour [17].

Much of the story told about the experience of healthcare workers in China draws attention to the negative consequences of financial incentives on worker motivation [18–20]. Often defined as ‘an individual’s degree of willingness to exert and maintain an effort towards organizational goals’ [19], the central issue regarding motivation through the use of financial incentives appears to rest on them becoming an end in themselves as they displace other types of reward (e.g. praise from supervisors or appreciation by the community) and challenge the public sector ethos and values of serving the interests of patients and the public.

The emerging consensus about healthcare worker motivation in China, as with other health systems, reflects the importance of ‘packaging financial and non-financial incentives’ [16]. In their review of health worker (HW) motivation in low and middle income countries (LMICs), Okello and Gilson [21] highlight how low levels of motivation have been linked to interventions focused exclusively on extrinsic methods such as pay-for-performance and monetary reward. Such approaches can also undermine the support and development of intrinsic motivation often associated with fulfillment at work, the quality of work performed, and the retention of staff. Okello and Gilson [21] summarise how intrinsic motivation often requires a different approach that is focused on the quality of social interactions, levels of autonomy and value alignment. Intrinsic motivation can also be influenced by fair treatment and respectful interactions between colleagues, supervisors, organisations and patients.

Research studying the interactions between healthcare reform and healthcare worker experience in China is clearly growing [15, 22]. Yet in depth qualitative accounts of these arrangements continue to be underdeveloped. Comparisons of healthcare worker experiences across different roles, organisations, and geographical areas warrant further inquiry. Moreover, there has yet to be an application of the motivation literature within the context of China’s healthcare reforms. Our paper appears to be the first in this regard in its application of motivation theories across different contexts and roles within China’s healthcare system. In this sense, it also provides a valuable contribution to the existing empirical evidence base regarding the key determinants of healthcare worker motivation. The following sections detail our aims and methods for the study before going on to present findings of healthcare worker motivation and reflecting on how such arrangements can be improved.

## Methods

Our research carried out comparative case studies of healthcare worker motivation across China. Based on the challenges faced by healthcare workers highlighted above, our aims were threefold in 1) analysing the interaction between financial incentives and healthcare worker motivation; 2) analysing the experiences of healthcare workers with regards to career development; and 3) analysing healthcare worker experiences of day to day practice.

To gain further in depth understanding of healthcare worker motivation, a qualitative research design was developed with the view to gaining an in-depth understanding of healthcare worker motivation. Given what we know about the importance of context in shaping healthcare worker motivation [21], as well as the vast range of contextual differences that can be found in China, the research design looked collect qualitative data from a range of experiences both geographically (differences across regions) and professionally (differences between roles). To do so, a purposeful sampling technique was used to identify and select individuals that were identified as especially knowledgeable about or experienced in relation to these research aims [23].

We carried out semi-structured interviews (*n* = 89) with a range of staff and organisations from across the regions of Shanghai (*n* = 22), Kaifen (*n* = 32), Urumqi and Shihezi (*n* = 35). The selection of these different geographical areas was done on the basis that these areas could capture any regional differences in terms of socioeconomic status of the population as well as capturing any differences in healthcare provider configurations. Within these areas our sample looked to gain healthcare worker experiences across a range of occupational groups and departments. The purposive sample of healthcare workers included doctors (*n* = 30), nurses (*n* = 35), and managers (*n* = 15) drawn from across tertiary and county hospitals, as well as primary care community health centres.

Our aim here was to undertake typical case sampling focusing on the shared experiences of the participants selected to interview [23]. The sample was obtained by approaching representative hospitals (the main tertiary and county hospital) and community health centres within each region. Recruitment was based on making contact with a senior manager or related official and providing them with information about our research. Following these discussions, we identified key doctors, nurses, and managers who were available and willing to participate. Given the practicalities regarding what was feasible within the timeframe of the research, the final interview numbers were based on a saturation point of obtaining a comprehensive understanding where no new substantive information was being acquired [24].

Semi structured interviews asked questions regarding healthcare worker experience. All participants were asked similar questions in relation to their income (What does your income include? What factors might affect your income?), their career development (Do you have a performance review system? Have you got any incentive strategies?), their reflection on the key issues facing the healthcare system (What do you think is the main problem in the current healthcare system? How could these be improved?), as well as wider reflections on their current role (What is important to you as a clinician/nurse/management?).

The interviews were carried out between June 2013 and March 2014. They were recorded subject to interviewee approval and lasted between 15 and 45 min. The research received ethical approval from Shanghai Jiaotong University Ethical Review Board. All participants were provided with a research information sheet about the study beforehand and asked to consider whether they were willing to participate in the study. Informed consent was provided by participants along with assurances that anonymity would be maintained by the research team.

The research carried out a thematic analysis of the transcribed interview text using the NVivo software. The data analysis process built on the analysis of all 89 interviews conducted and transcribed verbatim in Chinese. Coding and analysis of the data would initially build around the Chinese speaking authors reading the 89 interviews in Chinese to familiarise themselves with the data. Following these initial readings, discussions about potential themes were then developed and refined between the first author, second author, and the communication author. These subsequent themes were then translated into English (the second and communication authors are bilingual) as the thematic analysis proceeded through an iterative process of these authors revisiting the interview data, discussing, and refining this material.

The resulting themes focused on healthcare workers’ experience of financial incentives, career development, and day-to-day experience of meeting patient demand and meeting patient expectations. They were then analysed at an abductive level of analysis, cross matching the key insights from the qualitative data with established themes discussed in the healthcare worker motivation literature. For example, it became apparent that the various worker perspectives connected with individual, organisational, and social-cultural influences affecting day-to-day work as identified by Franco et al. [19]. On this basis, a process of abductive reasoning underpinned the data analysis in making connections between healthcare worker experiences and the motivation literature regarding income, career development, organisational tasks and responsibilities, and relationships between colleagues and the public.

## Results

Our research identified a range of motivational factors influencing healthcare workers in China. The section below captures these insights and presents them according to key themes related to income generation, performance review, meeting patient demand, and meeting patient expectations.

### Income generation

Our interviews presented a range of perspectives regarding the expectations and experience of healthcare workers. Central to these questions and concerns was a lack of extrinsic motivation where current salaried incomes were deemed insufficient to meet the basic needs of healthcare workers:

*I still hope that one day the principle of “to each according to his contribution” can be implemented. I hope that our income can become fairer and more reasonable to our position (Respiratory doctor)*

A perception prevailed healthcare workers were held in less regard compared to other professionals such as teachers and civil servants, as well as healthcare workers in other countries. Our interviewees described how low pay had led to perceptions of low-value and disrespect of their work:

*We cannot get the same salary as a teacher and civil servant might get despite us having the same educational experience as them. We cannot accept the fact that such an income gap exists with medical personnel (Head nurse)*

*Nowadays medical staff don’t get paid enough… In Taiwan, a chief surgeon can earn nearly 2,000,000 yuan a year, but here this is not possible for domestic doctors like me here (Doctor)*

Healthcare workers described how their basic salary was supplemented with additional payments. These supplementary payments were driven by fee for service procedures with amount received dependent on departmental performance measured by the number of patient encounters. These supplementary payments would be distributed between everyone in the same department.

Our interviews found that these additional payments proved to be divisive as healthcare workers described how they were more relevant to tertiary teaching hospitals rather than other parts of the healthcare system. It was only these hospitals that were able to generate this additional income through teaching subsidies and involvement in research activity where supplements were awarded for publication in national and international journals.

Our research found that senior doctors, or the ‘top 10%’, demonstrated what could be described as higher-level motivations and goals in terms of fulfillment and satisfaction. This was in large part due to their ability to access extra money by seeing more patients, participating in research, and occupying higher price fee for services.

*If doctors get research funding or publish a paper, the hospital will give them some bonus. For example, if a doctor successfully wins a national research project, the hospital gives them 30,000 yuan. (Radiation oncologist)*

Frustrations regarding limited access to income were expressed by those working outside of these settings, particularly from those lower in the professional hierarchy. Nurses were highly critical of the current arrangements. With limited access to other forms of income, they expressed frustrations at the pay gap between themselves and doctors.

*Our hospital and our department encourage us to publish papers and apply for funding. But it’s very difficult for nurses to accomplish these tasks alongside our other tasks whereas doctors can fulfill these tasks (Ward nurse)*

Junior Doctors expressed frustrations at the inequities linked to the current fee for service arrangements. High workloads and lack of training meant they did not have the time or the ability to get involved in research activity.

*I think there is problem with the income system… we have a lot of pressure to maintain our performance otherwise we don’t get any bonuses. I hope they can improve the incentive system… [at the moment] it seems that there is nothing else except the bonuses from publishing papers (Cardiologist)*

The financial incentives were such that those hospitals delivering routine services in specialist tertiary care received more payment compared to other hospitals. Outside of tertiary settings there were not the same opportunities for other hospitals or for those delivering primary care:

*We have limited grey [supplementary] income here since it’s such a small hospital. Of course, this is different for the other provincial hospitals which have more patients and more activity (Middle manager)*

The income gap appeared to be most pronounced when describing the arrangements in primary care. A ‘huge gap’ existed between hospital care and primary care. Community Health Centres (CHCs) were a case in point. Relying solely on funding from local government, they were unable to reap any financial benefits from additional funding streams. Furthermore, the preference for people to go to hospitals rather than primary care meant CHCs tended to treat only patients with chronic conditions whose requirements did not have the same incentives and rewards as routine operations found in acute care settings.

*GPs need to be on call 24 hours a day. Residents might call us for a service even if they have a cold, no matter how late… but we have no bonus… (GP)*

### Performance review

Connected to the issues of income generation outlined above, our research found healthcare worker motivation to be shaped by having insufficient performance appraisal systems in place. With salaries and financial rewards built around the quantity of outputs as measured by patient admissions, opportunities for career progression would depend on workload rather than individual performance, measures of positive patient experience, or service quality. As it stood, healthcare workers described how performance review equated to ‘quantity of work accomplished’:

*We still don’t have incentives in place. It depends on the overall development and performance of the hospital. Everybody knows that if the hospital performs well, they get the income not staff (HR Manager)*

*We don’t really have a performance review system. It doesn’t matter how much you do or don’t do. If the department performs well, everybody gets the same bonuses equally. (Head nurse)*

The picture presented was one where to get promotion or to develop a career, additional activity was needed. Promotion was connected to additional research activities that were out of reach for the majority of staff, particularly nurses who experienced frustrations with the limited opportunities for career development.

Yet despite these criticisms, there were some suggestions that the situation was changing. Improvements to working conditions were noted with the arrival of new buildings and better infrastructure. Healthcare workers described how their organisations were starting to introduce development plans, with questions and criteria of assessment being introduced that went beyond solely the number of patients being processed:

*We have never really had a performance review system but now we are building one where the main criteria will be both quantity of work and quality of work accomplished (Ward nurse)*

### Meeting patient demand

Healthcare workers described how recent reform efforts led to increases in patient demand. The combination of significant increases in those with insurance, combined with reductions in drug costs, had meant that demand for services increased rapidly. A number of those interviewed described the positive effects of these reforms for patients with the expansion of medical insurance schemes increasing healthcare coverage for the population:

*Before the reforms patients had to apply for reimbursements from their workplace. Now they can see a doctor with their medicare cards and pay a proportion of the reimbursement. It’s much better for them (Chinese traditional medicine doctor)*

However, such a dramatic increase in patient interactions was creating significant strain on the workforce. Supply was not able to keep up with demand as reforms intending to increase access to services also increased workload.

*Now more and more people have medical insurance so they are more inclined to go to large hospitals. As a result, the workload is larger than before… doctors are now having to work 10 hours or more every day (CT doctor)*

There were concerns raised at the low cost for certain procedures which was leading to an increase in demand for certain services:

*Private hospitals can charge $1000 in foreign countries for a CT scan. Here, the cost is low. A domestic CT scan is just 200 yuan, which includes the report, diagnosis, and feedback. (Doctor)*

While increasing demand represented a shared theme across the occupational groups, the theme was most pronounced for those working in large secondary and tertiary hospitals. Central to the demand issue was patient preferences to go to tertiary hospitals for all types of care. This placed particular pressures on these providers:

*Nowadays there are far too many patients in big hospitals (secondary and tertiary care hospitals) and doctors are under tremendous pressure. My hospital is in Kaifeng, which is quite close to the provincial capital Zhengzhou… Given the very convenient location patients come here for all kinds of illnesses however minor (CT Doctor)*

*There is a big difference between primary care and tertiary care. Our government wants to design a system that is similar to other countries, that is, where patients go to their GP first and then go to the referral system if needed. But in reality, it’s not possible here as patients go straight to tertiary hospitals (Chinese traditional medicine doctor)*

With healthcare workers painting a picture of high demand for tertiary hospital care, the opposite was apparent for those working in primary care. Here the nature of patient demand was different with those working in these contexts describing the delivery of care to predominately older patients with chronic conditions.

*Patients with chronic diseases normally come to primary care… GP practices are close to where they live and more convenient for them. Our patients are mostly above 60 years old… Patients under 40 years old wouldn’t choose us.. they go to big hospitals (secondary and tertiary care hospitals) for any kind of disease. (GP)*

Describing the nature of patient demand as being significantly less in primary care, GPs expressed frustration at the range of laborious tasks associated with primary healthcare services. These roles described how many of the performance indicators that were introduced were not applicable to these primary care contexts. The recent reforms to primary care had created unnecessary bureaucracy:

*There are too many indicators for us to fulfill… such as occupancy bed rates and prescription drug use rates. According to the regulations, GPs now need to sign up 500 families every month no matter how many people there are in the community or whether the residents actually want to sign up (GP)*

### Meeting patient expectations

Faced with the difficulties of meeting patient demand, our research identified wider cultural questions regarding the challenges of maintaining relationships with patients. Lack of time and space led to communication breakdowns with patients. Safety fears were also raised as the pressure to process patients made them susceptible to inappropriate medical procedures. These high pressure and high risk situations were leaving healthcare workers exposed to the threat of violence and abuse from patients unhappy with the service provided. Numerous reports were cited of violence towards staff as the result of disagreements and objections to the care being received:

*Doctors need to examine so many patients everyday… it means sometimes they don’t have the time for deep communication… unfortunately it leads to patients feeling dissatisfied which is difficult for us (Doctor)*

As the workers with most contact with patients, nurses in particular suggested that they were concerned about their safety at work:

*Nowadays patients are fierce and we are all scared of them. Sometimes [they] abuse us but we have to tolerate them… Even if there is a tiny problem they can try for compensation money or even worse act in a violent way (Clinic nurse)*

Accounts of verbal and physical abuse were also connected to a lack of respect and authority displayed to healthcare workers. The low status of doctors and nurses meant they were vulnerable to abuse and violence as patients questioned their judgement and authority. There was not a ‘harmonious relationship’ between healthcare workers and patients in large part due to a breakdown in trust relationships. More work was therefore needed to build trust and improve patient awareness and education across the population.

*No matter what happens, it’s always the doctors’ fault. The problem is that society as well as patients pay little respect for doctors. (Doctor)*

*I really hope we can get more reward and recognition in the future. We’re not asking for a lot, but at least our safety should be guaranteed at work. (Cardiologist)*

## Discussion

Our research has found that the financial and performance related incentives underpinning healthcare reform efforts are negatively impacting on the healthcare workforce. This experience appears to be a common one as displayed by the relatively minor, if any, differences of opinion across the areas we studied. Our interview data finds a marginal increase in emphasis on supplementary income activity across the eastern province of Shanghai, however, a central theme running throughout our findings is the shared experience of healthcare workers across these contexts. In the following section we reflect on these developments and highlight ways in which China can improve the motivation of healthcare workers.

### Improving motivation

The accounts of our healthcare workers in China highlight how a healthcare system reliant on the use financial incentives can challenge the ability of those working in it to maintain the values and ethos of public service.

Central to the issues facing healthcare workers is the disparities in pay as income generation has become largely dependent upon access to supplementary income streams in the form of higher end fee for services and research publication revenues. To reduce these disparities, our findings suggest that an increase in the extrinsic income of staff is needed to better reflect the contribution of different workers across the healthcare system. To do so we suggest that the current fee for service funding arrangement, which appears to be only benefiting large tertiary hospitals, needs to be modified.

Drawing on the experience of other countries, our findings suggest that China should look to other forms of payment for performance to promote equity. The introduction of capitation payment methods promoting population based service delivery represents a clear opportunity in this regard. Experiments of this payment method in China appear to show positive results in its ability to nudge behaviours of the healthcare workforce away from the ‘for profit’ mind set [25]. Moving to such a payment method could also bring a range of ‘softer’ benefits through the creation of better working environments that promote greater collaboration and team working rather than competition and resource accumulation.

Our findings also suggest that other extrinsic options should be explored in order to close the gap between worker performance and financial reward. The restructuring of salary scales to create benchmarks across the public sector represents a possible option for policy makers to create greater transparency and openness about career progression. Other options for healthcare workers might include the introduction of a living wage; the creation of public sector benefit packages such as greater holiday; a review of current pension arrangements; or the creation of reduced insurance contributions for healthcare workers.

Alongside these extrinsic options, our findings suggest that significant work is needed to improve the intrinsic motivation of staff. Many of our interviewees expressed a commitment to their job but low morale caused by perceptions of low status and limited career options were clearly apparent. Frustrations at the lack of promotion opportunities suggest further work is needed to develop continuous career and professional development opportunities for all staff. A greater emphasis on human resource management (HRM) practices could provide practical options here in the promotion of training and development, the importance of teamwork, and the development of leadership for quality and service improvement.

Such findings add further weight to the evidence obtained elsewhere that to achieve high performing work systems in healthcare requires investment in HRM practices that are able to recruit, develop, motivate and retain employees [26–28]. Such a relationship between HRM activities and organisational performance is well documented. Leggatt et al. [27] for example summarise evidence from elsewhere that psychological empowerment is positively associated with perceptions of the quality of patient care. West et al. [29] suggest that greater use of a HRM practices has a statistically and practically significant relationship with patient mortality. Their results suggest that people management systems that emphasize a set of complementary ‘high involvement’ policies and practices (i.e., an emphasis on training, performance management, participation, decentralised decision making, involvement, teams, and employment security) are successful in contributing to high-quality healthcare [30].

China could also look to current interest in leadership and Organisational Development initiatives to provide possible mechanisms which have been shown to provide intrinsic benefits for healthcare workers [31]. Given the context and history of China, the implementation of any such initiatives would require further translation to make them conducive to these healthcare contexts, however, the views of interviewees in our research suggest that new conversations and framings of healthcare reform would be welcome.

#### Building trust

Our findings highlight how healthcare workers in China are struggling to meet the pressures of ever increasing patient demand. With rising tensions and anxieties in the face of patient violence and unrest, the need to improve relationships between the workforce, patients and the public was clearly highlighted.

These findings reflect those identified elsewhere [9] showing how poor interpersonal relations between HWs and patients have been attributed to staff shortages and heavy workload. The challenges of meeting patient demands and managing patient expectations are indicative of research [21] pointing to how a breakdown in trust relationships is often associated with divisions of labour and strained relations between occupational and societal groups. Our research highlighted how nurses in particular did not feel appreciated, with a lack of fairness regarding their current position being conveyed. GPs also conveyed a breakdown in trust relationships as government policy became synonymous with bureaucratic procedures and inadequate financial reimbursement.

To improve relationships and build trust between healthcare workers, patients and the public our findings point to the need for change to the ‘patient output’ culture that preoccupies the Chinese healthcare sector. Changes to the dynamics of these relationships can be achieved by providing opportunities for feedback and communication between workers and patients. Exploring a greater role for third parties such as managers, patient and staff representatives, or third party organisations to facilitate mediation, raise concerns and resolve conflicts should also be considered. Such interactions and conversations support calls from elsewhere regarding the need to develop ‘soft’ intelligence in healthcare organisations seen as central to the development of trust relationships [32]. Initiatives that encourage greater responsibility, respect and appreciation from patients also warrant consideration [33]. Further education regarding health conditions, the role of primary care, and the need for respect and tolerance of staff represent important next steps for China.

The experience of China reflects what has been documented elsewhere that to achieve a motivated workforce requires a multi-channel approach. Where much of China’s healthcare reform efforts have been extrinsically based, further research is needed to develop the intrinsic dimensions of trust and motivation across the healthcare workforce. In doing so, China learn from elsewhere in terms of further understanding the needs of its workforce. Comparative workforce analysis could provide valuable insights into ways in which China is similar or different to other low, middle and high income countries. Benchmarking of worker motivation and performance through the application of methodologies and metrics that have been developed elsewhere (e.g. NHS staff survey) might provide a fruitful line of inquiry.

Evidently there are a number of limitations with our research. The ability to compare and contrast these qualitative experiences with quantitative measures either in the form of cross sectional survey data or secondary data concerning actual income and HR practices is lacking. The nature of the purposive sample could have missed out important perspectives that warrant further analysis e.g. the perspective of rural healthcare workers. We encourage future mixed methods research in this area in order to better understand and triangulate these experiences. We also encourage research that provides further in depth qualitative accounts of these particular themes concerning workforce motivation. Our research provides an important map from which to build theoretical and empirical research in this area.

## Conclusion

China’s healthcare reform efforts continue to receive much attention. Our study represents a first in connecting the experiences healthcare workers in China with wider discussions regarding healthcare worker motivation across the globe. Through its analysis of healthcare worker experiences across a range of contexts, it has documented how worker motivation in China is being influenced by the intended and unintended consequences of extrinsic financial incentives. With the aim to help understand these issues as well as look at ways to improve these efforts, our paper has introduced possible solutions to assist policy makers in resolving these issues.

In conclusion, our paper suggests that multi-channel approach is needed to improve healthcare worker motivation. We recommend the further development of policy and practice that recognises the importance of communication, organisational development and human resource management. By drawing attention to this broad range of influences, our paper aims to help policy makers in the development of reform programs that can effectively promote worker motivation, and hence, improve the healthcare system for all those involved.
